# Effect of Emamectin Benzoate on Root-Knot Nematodes and Tomato Yield

**DOI:** 10.1371/journal.pone.0141235

**Published:** 2015-10-28

**Authors:** Xingkai Cheng, Xiumei Liu, Hongyan Wang, Xiaoxue Ji, Kaiyun Wang, Min Wei, Kang Qiao

**Affiliations:** 1 College of Plant Protection, Shandong Agricultural University, Tai’an, Shandong People’s Republic of China; 2 Cotton Research Center, Shandong Academy of Agricultural Sciences, Jinan, Shandong, People’s Republic of China; 3 Plant Protection and Inspection Station of Feicheng, Feicheng, Shandong, People’s Republic of China; 4 College of Horticulture Science and Engineering, Shandong Agricultural University, Tai’an, Shandong, People’s Republic of China; Northwest A&F University, CHINA

## Abstract

Southern root-knot nematode (*Meloidogyne incognita*) is an obligate, sedentary endoparasite of more than 3000 plant species, that causes heavy economic losses and limit the development of protected agriculture of China. As a biological pesticide, emamectin benzoate has effectively prevented lepidopteran pests; however, its efficacy to control *M*. *incognita* remains unknown. The purpose of the present study was to test soil application of emamectin benzoate for management of *M*. *incognita* in laboratory, greenhouse and field trials. Laboratory results showed that emamectin benzoate exhibited high toxicity to *M*. *incognita*, with LC_50_ and LC_90_ values 3.59 and 18.20 mg L^-1^, respectively. In greenhouse tests, emamectin benzoate soil application offered good efficacy against *M*. *incognita* while maintaining excellent plant growth. In field trials, emamectin benzoate provided control efficacy against *M*. *incognita* and resulted in increased tomato yields. Compared with the untreated control, there was a 36.5% to 81.3% yield increase obtained from all treatments and the highest yield was received from the highest rate of emamectin benzoate. The results confirmed that emamectin benzoate has enormous potential for the control of *M*. *incognita* in tomato production in China.

## Introduction

Southern root-knot (*Meloidogyne incognita* (Kofoid and White, 1919) Chitwood, 1949) is one of the most economically important plant-parasitic nematode species that can attack the roots of more than 3000 agricultural crops. *M*. *incognita* plays a vital role in the predisposition of the host plant to invasion by secondary pathogens, resulting in significant yield losses [1–4]. In Shandong, China, a large vegetable producing province, root-knot is an important disease [5]. About half of greenhouse-grown vegetables are infected by *M*. *incognita* with an annual loss estimated to be more than $400 million [6].

Over the last few decades synthetic nematicides, especially the fumigant nematicides, have been the most important means to control *M*. *incognita*, but their uses have recently been restricted. Many products (e.g., methyl bromide) are no longer available to growers because they affect nontarget organisms and may harm the environment [7,8]. Many chemical alternatives and their combinations have been suggested as methyl bromide alternatives [7]. 1,3-Dichloropropene and chloropicrin are the most common fumigant alternatives adopted, followed by metham sodium and dazomet [8]. Due to the regulatory constraints and public resistance to fumigant use, development of a viable fumigant-free alternative to control *M*. *incognita* is needed.

Non-fumigant tactics to control root-knot nematodes, such as soil solarization [9], grafting [10], organic amendments [11], and biocontrol agents have been evaluated [12]. The Chinese Government is committed to developing biological pesticides. Botanicals are often considered a substitute to chemical pesticides [13,14]. Caboni et al. [13] reported the nematicidal activity of mint aqueous extracts against *M*. *incognita*, and the results showed that mint species containing reactive carbonyl compounds had potential use as bionematicides.

Emamectin benzoate is a biological insecticide derived from naturally occurring avermectin molecules isolated by fermentation from the soil bacteria *Streptomyces avermitilis* Kim & Goodfellow [15]. Emamectin benzoate acts as an antagonist for gamma-aminobutyric acid-gated chloride channels, which causes disruption of nerve impulses and rapid paralysis in a range of lepidopteran species [16,17]. Traditionally, emamectin benzoate is applied as foliar spray to control lepidopteran insect. But recently its application to soil has become more widely used. Soil application has more benefits than foliar application, such as being less hazardous to applicators and having lower detrimental effects to natural enemies of insects [18]. Data on efficacy of emamectin benzoate against arthropods are often obtained from application as foliar sprays, but according to some research, emamectin benzoate also has potential as a soil nematicide [19].

Therefore, repeated laboratory, greenhouse and field trial tests were conducted to assess soil application of emamectin benzoate for management of *M*. *incognita* and to determine its effects on tomato yield.

## Materials and Methods

### Chemicals

Emamectin benzoate (>95% pure, Syngenta, China) was dissolved in acetone to various concentrations (200, 100, 50, 25, 10, 5.0 and 1.0 mg a.i. L^-1^). Emamectin benzoate (1% EC, a.i., Shandong Luba Chemical Co., Ltd., Jinan, China). Cadusafos (92% pure and 10% G, FMC Corporation, Chicago, IL), which is still available to the local farmers to control nematodes, was included as a standard treatment for comparison. Technical materials were used in the laboratory test and pesticide formulations were used in the greenhouse and field trials. All reagents and solvents were of pesticide grade.

### Laboratory test

Perineal configuration, esterase electrophoretic pattern and host range analyses were used to identify the isolated nematodes as *M*. *incognita* [20]. It was originally isolated from tomato plants in Shandong and maintained on tomato (cultivar “Chaoqun Fenguan F_1_”) roots at the greenhouse in Shandong Agricultural University. For these experiments, all plants were maintained in a growth chamber at 25±2°C, 60% relative humidity with a light: dark (16: 8h) photoperiod, in plastic pots (20 cm diameter). Plants used for inoculations were 8 weeks old. After 45 days, the plants were removed from the pots, and the roots were washed free of soil and cut into 2 cm pieces. Eggs were extracted with sodium hypochlorite (NaClO) procedure, and second-stage juveniles (J2) were allowed to hatch in modified Baermann funnels at 25°C [21]. All J2 hatching in the first 3 days were discarded, and thereafter J2 collected after 24 h were used in the experiments.

The nematicidal efficacy of emamectin benzoate and cadusafos against J2 of *M*. *incognita* was determined in aqueous tests. Emamectin benzoate and cadusafos treatments (200, 100, 50, 25, 10, 5.0 and 1.0 mg a.i. L^-1^) were prepared in acetone + distilled water (10: 90% by volume), and distilled water, as well as a mixture of water with acetone at concentrations equivalent to those in the treatment wells, were used as controls. Then 1 mL of solution and 1 mL of root-knot nematodes J2 (containing average 150 J2) was added to each well of a 24-well plate. Well plates were wrapped with parafilm^®^, placed in plastic zip-lock^®^ bags and stored in aluminum foil pans covered with another pan to keep them dark. Units were kept at 25°C. After 48 h, the relative percentages of the motile and immotile J2 were evaluated using an inverted microscope (Olympus, China) at 40×magnification. Furthermore, nematodes were moved to distilled water after washing in tap water through a 20 μm pore screen to remove excess chemicals. To confirm the nematicidal activity of emamectin benzoate, immobile 30 J2 from each treatment were collected from the above experiments, transferred to tissue culture plates filled with water, and monitored for 12 h. The experiments had five replications and were repeated three times.

### Greenhouse tests

A population of *M*. *incognita* originally obtained from tomato roots collected from a greenhouse in Shandong Agricultural University, Tai'an, Shandong, China. All plants were maintained in a growth chamber at 26–28°C, 60% relative humidity with a 16: 8 h light to dark photoperiod. Greenhouse experiments were conducted in 12 cm square pots containing a silt loam soil collected in a commercial field near Fang country. The organic matter content of the soil was 19.5–24.8 g kg^-1^, the bulk density was 1.2 g cm^-3^ and the pH was 7.1–7.3.

Pots were watered before transplanting and 40 day old tomato seedlings of uniform growth were selected and transplanted. After that, 2000 J2 were inoculated into holes surrounding the root. Tomatoes were treated 3 days after transplanting. Emamectin benzoate (1% EC, a.i.) was applied to the soil at 150, 100 and 75 g ha^-1^ and cadusafos (10% G, 8.0 kg a.i. ha^-1^) was also applied to the soil. The experiment was repeated with each treatment replicated 10 times. The pots were watered as needed.

### Field trials

Two field trials were carried out in a commercial tomato field near Fang country, Tai’an city, Shandong, China. The field trials were established in summer 2014 and spring 2015, respectively. Tomatoes had been grown on the selected farm for 12 years and no fumigants were used previously. The soil was a silty loam, with an organic matter content between 22.1 g kg^-1^ soil, a pH of 7.1–7.3 and a bulk density of around 1.2 g cm^-3^. The selected experimental site had a history of high population *M*. *incognita* (54 J2/100 mL of soil). Before planting bed formation, the plots were disked twice.

Treatments were arranged in a randomized block design with five replications. Chemical treatment doses were based on label application directions. Treatments were: (a) emamectin benzoate (1% EC, a.i.) furrow applied at a dose of 150 g ha^-1^; (b) emamectin benzoate furrow applied at a dose of 100 g ha^-1^; (c) emamectin benzoate furrow applied at a dose of 75 g ha^-1^; (d) cadusafos (10% G, a.i.) furrow applied at a dose of 8.0 kg ha^-1^, and untreated control.

Individual plot consisted of five rows with plots size of 30 m^2^, and there were approximately 120 tomato plants per plot. Every plot was irrigated with 1.5 cm water per block separately to avoid cross contamination the day before chemicals were applied for better bedding. On the day of chemical application (July 16, 2014), emamectin benzoate and cadusafos were applied in the furrow 0.25 m deep and 0.50 m apart just on the planting rows. The planting rows were bedded and pressed 0.80 m wide at the base, 0.70 m wide at the top, 0.20 m high and spaced 0.75 m apart on centers.

Six-week-old ‘Chaoqun Fenguan F_1_’ tomato seedlings were transplanted into the top of the beds after chemical application. Raised beds were 1.5 m apart and each contained 20 tomato plants spaced 0.50 m apart in the row. Plants were staked and tied as needed during the season. Flood irrigation was provided according to the water requirements of the crops. Insecticides and fungicides were applied weekly beginning three weeks after treatment (WAT) following current recommended practices.

### Data analysis

All the data were analyzed for homogeneity of variances. When the variance was equal, the pooled data of tests were combined.

Data from the laboratory tests were analyzed by logit/probit dose response/mortality regression calculated using SPSS probit procedure (SPSS, version 15.0). Adjusted mortality was calculated using Schneider-Orelli’s formula, whereby mortality was calculated as a percentage and adjusted to mortality in the control (solvent only) using the equation: %mortality adjusted = 100×[(% mortality treated-mortality control)/(100-mortality control)] [22]. Adjusted mortality was used to calculate lethal concentrations (LC) required to kill 50% (LC_50_) and 90% (LC_90_) of nematodes.

For the greenhouse tests, plant heights were measured from 10 plants at 30 days after transplanting (DAT). Root-knot nematode infection was determined at the same time by carefully removing roots and rating the roots for nematode galls on a scale of 0–10, where 0 = no galls, 1 = 0–10% of roots galled,… up to 10 = 90–100% [23]. After gall rating analysis was performed, root fresh weight was recorded.

For the field trials, plant heights were determined from 10 plants per plot at 30 and 50 DAT. *M*. *incognita* populations were counted at 20, 40, 60 DAT by extracting soil samples with a soil probe (2.5 cm wide by 20 cm deep) from the rhizosphere of 10 tomato plants per plot, and extracted from 100 cm^3^ soil using a standard sieving and centrifugation procedure for counting [24]. Root galling index was determined at 14 WAT by digging the roots of 10 plants per plot and evaluating root damage as described above. Nematode control effect was calculated using the formula: [(Root galling index control- Root galling index treated)/ Root galling index control]. Marketable tomato fruits were harvested twice at 12 and 14 WAT, and graded into the extra-large, large and medium categories.

Prior to analysis, data expressed as percentages were arcsine transformed to homogenize variances. Sources of variation were treatments and blocks. The effects of different chemical treatments were examined using analysis of variance (ANOVA) and when the *F*-test was significant at *P* < 0.05, treatment means were compared using the Student-Newman-Keuls test (SPSS, version 15.0 for Windows).

## Results

### Laboratory test

**Fig 1:**
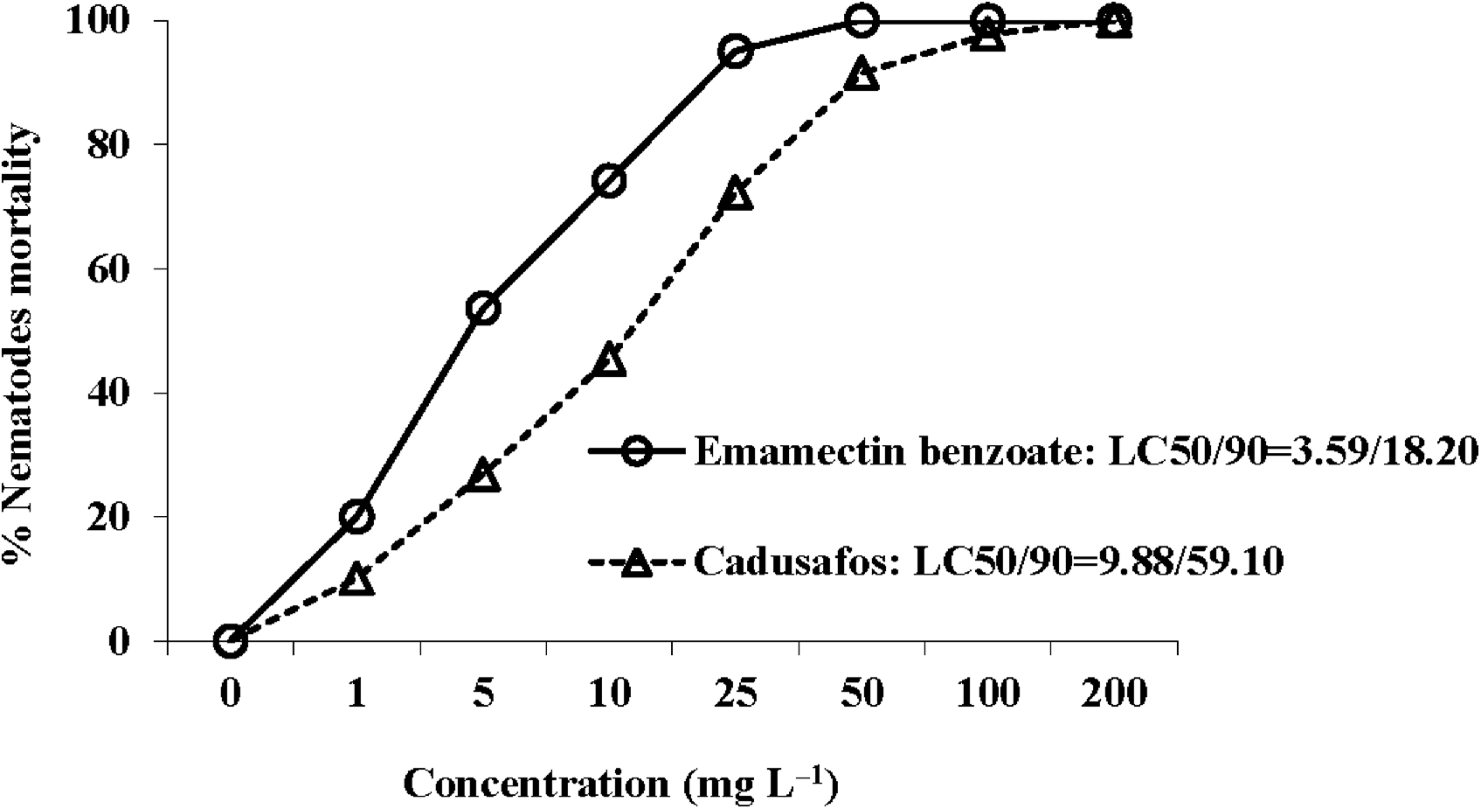
Nematode mortality (%) and lethal concentrations (LC_50_ and LC_90_) (mg L^-1^) of emamectin benzoate and cadusafos against *Meloidogyne incognita* (Kofoid and White, 1919) Chitwood, 1949 juveniles (J2, after 48h).

None of the immobile J2 recuperated in water, proving that emamectin benzoate tested act as a nematicide. Emamectin benzoate at the rate of 25 mg ai L^-1^ caused >70%mortality of nematode J2 (Fig 1). The calculated LC_50_ and LC_90_ after 48 h for J2 were 3.59 and 18.20 mg ai L^-1^ for emamectin benzoate as opposed to 9.88 and 59.10 mg ai L^-1^ for cadusafos (Fig 1).

### Greenhouse tests

All rates of emamectin benzoate significantly reduced root-gall index on tomato while keeping good plant growth (Table 1). The highest plant height (45.3 cm) was realized in 150 g ha^-1^ emamectin benzoate and cadusafos treated plots. Other treatments had moderate height, better than the untreated control infected with *M*. *incognita*. Tomato fresh root weight had a similar trend as plant height. Tomatoes grown in the untreated plots had the highest root galling index (5.78). Treatments involving emamectin benzoate and cadusafos provided a 57.6–87.7% *M*. *incognita* control efficacy compared with the untreated control.

**Table 1:** Effects of emamectin benzoate and cadusafos on plant height, fresh root weight and *M*. *incognita* ^a^ root galling in the greenhouse.

Chemicals	Dose (ha)	Plant height ^b^ (cm)	Fresh root weight (g)	Root galling index ^c^	Nematode control effect ^d^ (%)
Emamectin benzoate	75 g	37.3ab	1.11 b	1.54 c	73.4
Emamectin benzoate	100 g	43.1 a	1.41 a	1.05 cd	81.8
Emamectin benzoate	150 g	45.3 a	1.53 a	0.71 d	87.7
Cadusafos	8.0 kg	36.7 b	1.04 b	2.45 b	57.6
Control without nematodes	-	35.1 b	1.02 b	-	-
Control with nematodes	-	32.1 c	0.87 c	5.78 a	-

^*a*^ *M*. *incognita = Meloidogyne incognita* (Kofoid and White, 1919) Chitwood, 1949.

^*b*^ Plant height and fresh root weight was determined at 30 days after transplanting (DAT). Data are arithmetic means of ten replications and

means separated with Student-Newman-Keuls test (*P* < 0.05).

^*c*^ Nematode root galling index determined at 30 DAT obtained using a 0–10 scale where 0 = no galls and 10 = 90–100% of roots galled. Data transformed with arc sine square root and means separated with Student-Newman-Keuls test (*P* < 0.05).

^*d*^ Nematode control effect was calculated using the formula: [(Root galling index control- Root galling index treated)/ Root galling index control].

### Field trials

Emamectin benzoate and cadusafos application significantly affected plant height, where ratings were increased compared to untreated controls (Table 2). In both experiment, the highest plant heights were achieved in the 150 g ha^-1^ emamectin benzoate treated plots. Other treatments had intermediate height, better than the untreated control. Moreover, there was a significant positive relationship between plant heights and emamectin benzoate doses.

**Table 2:** Effect of emamectin benzoate and cadusafos on plant height, nematodes control and tomato marketable yields in two field trials.

Chemicals	Dose (ha)	Plant height ^a^ (cm)			Nematodes in 100 cm^3^ soil ^b^		Root galling index ^c^	Tomato marketable yield (t ha^-1^)			
		30 DAT	50 DAT	20 DAT	40 DAT	60 DAT		Extra-large	Large	Medium	Total
Emamectin benzoate	75 g	41.8 ^d^ b	76.1c	23.5b	24.6b	26.5b	3.58b	5.4bc	17.3b	28.5b	51.2bc
Emamectin benzoate	100 g	44.1b	87.8b	20.5bc	20.3bc	21.1bc	2.67bc	6.3b	17.4ab	32.8ab	56.5b
Emamectin benzoate	150 g	46.3a	92.1a	14.2c	12.3c	11.5c	1.54c	7.1 a	20.8 a	40.1 a	68.0a
Cadusafos	8.0 kg	42.3b	74.3c	25.3b	26.8b	27.5b	3.45b	6.1b	18.1ab	33.2ab	57.4b
Control	-	32.6c	60.4d	43.3a	51.2a	57.4a	5.18a	3.5c	12.6c	21.4c	37.5d

^*a*^ Plant height was determined from 10 plants per plot at 30 and 50 days after transplanting (DAT) in the two field trials.

^*b*^ Nematodes (*Meloidogyne incognita* (Kofoid and White, 1919) Chitwood, 1949) in 100 cm^3^ soil were counted at 20, 40 and 60 DAT using a standard sieving and centrifugation procedure in both growing seasons.

^*c*^ Nematode root galling index determined at 14 WAT obtained using a 0–10 scale where 0 = no galls and 10 = 100% of roots galled.

^*d*^ Data are arithmetic means of ten replications and means separated with Student-Newman-Keuls test (*P* < 0.05). Numbers in the same column followed by the same letter are not significantly different according to Student-Newman-Keuls test (*P* < 0.05).

In both trials, *M*. *incognita* was isolated but other species of nematodes were below detectable level. The field results consistent with the results obtained in the laboratory and greenhouse tests, which proved that emamectin benzoate was a good nematicide. Treatments involving emamectin benzoate and cadusafos were effective in lowering population levels of *M*. *incognita*. Tomatoes grown in the untreated plots had the greatest number of nematodes and the highest root galling index. In contrast, emamectin benzoate at the maximum dose was the most effective treatment for reducing galling from *M*. *incognita* (Table 2).

All the treatments increased the marketable crop yields compared with untreated control. In the tomato crop, there was a 36.5% to 81.3% yield increase from the various treatments compared with the control (Table 2). The highest yield of extra-large fruit (7.1 t ha^-1^) was achieved in the emamectin benzoate at the maximum dose, while the lowest was obtained in the untreated control (3.5 t ha^-1^). Other treatments provided 5.4 and 6.3 t ha^-1^ yield within the same fruit category. A similar trend was observed for total marketable fruit yield, where the highest yield (68.0 t ha^-1^) was produced in the emamectin benzoate at the maximum dose.

## Discussion

### Advantage of emamectin benzoate as a biological pesticide

Biological pesticides have attracted considerable interest in recent years [25–28]. The attraction of using biological pesticides is the cost effectiveness of their production and environmentally friendly. In China the government is encouraging the development of biological pesticides [29]. Emamectin benzoate is a novel insecticide derived from the natural products in the avermectin family with improved thermal stability, greater water solubility, and a broader spectrum of insecticidal activity than those of avermectin [30]. On the other hand, emamectin benzoate has been mostly applied as a foliar spray, soil application has not been commonly practiced. The further development of emamectin benzoate for the huge market in China has great potential to reduce the effect that root-knot nematodes plays in the production of many crop plants.

### Basic of biology of emamectin benzoate to control *M*. *incognita*

A set of laboratory, greenhouse and field trial tests demonstrated the potential of emamectin benzoate as a soil application to control *M*. *incognita* and improve the yield of tomato. Emamectin benzoate is a very good nematicide, better than that of a standard nematicide cadusafos [7,31]. Emamectin benzoate caused irreversible paralysis on root-knot nematodes and the lowest effective dose to kill *M*. *incognita* was 0.1–0.5 mg kg^-1^, less than that reported for abamectin [32].

The greenhouse tests and field trials results confirmed the results from Ding et al. [19], who reported that emamectin benzoate was a promising nematicide for the control of root-knot nematodes in tomato. When the 500 and 1000 times dilution of emamectin benzoate were used, the relative control efficacies were 93.67% and 79.69%. Tomato yields increased by using emamectin benzoate in two field trials. There was a positive relationship between emamectin benzoate dose and tomato yield. Also, there was no phytotoxicity observed in the greenhouse test. All results indicate that emamectin benzoate has great potential as a soil nematicide.

In addition, emamectin benzoate is sensitive to light and has a rapid degradation rate in natural conditions so that its biological activity was greatly limited in the fields [33]. Emamectin benzoate degrades in soil which suggests that split applications may improve nematode control. On the other hand, slow release formulations of emamectin benzoate (e.g., microencapsulation) may solve the problem [32].

In conclusion, the results of this study demonstrated that emamectin benzoate exhibited a high nematicidal activity on *M*. *incognita* and increased marketable tomato yields, which suggested that emamectin benzoate is an excellent nematicide. However, further studies concerning prolonging its effective duration, promotion mechanism, dose and time of application after transplanting for a better performance in the tomato crop.

## Supporting Information

S1 Fig Graphical abstract. (PDF)
