# Supplementary figures and images for: Effect of Emamectin Benzoate on Root-Knot Nematodes and Tomato Yield
Source: PLoS One. 2015 Oct 28;10(10):e0141235. doi: 10.1371/journal.pone.0141235 (PMC4624971; doi:10.1371/journal.pone.0141235)

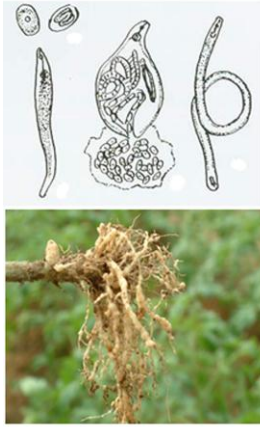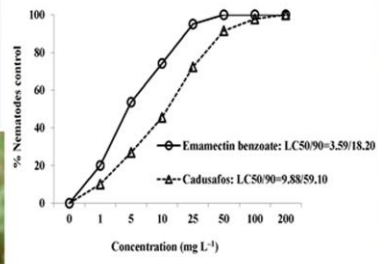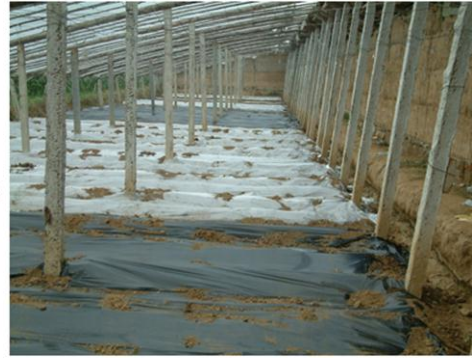

Supplement: S1 Fig — (PDF) [file pone.0141235.s001.pdf]
